# Methyl 4′,5-dichloro-2-hy­droxy-4,6-dimethyl­biphenyl-3-carboxyl­ate

**DOI:** 10.1107/S1600536812012676

**Published:** 2012-03-28

**Authors:** Muhammad Adeel, Peter Langer, Martin Hein, Helmut Reinke

**Affiliations:** a Gomal University, Department of Chemistry, Dera Ismail Khan (KPK), Pakistan; b Universität Rostock, Institut für Chemie, Abteilung für Organische Chemie, Albert-Einstein-Strasse 3a, 18059 Rostock, Germany; c Universität Rostock, Institut für Chemie, Abteilung für Anorganische Chemie, Albert-Einstein-Strasse 3a, 18059 Rostock, Germany

## Abstract

In the title compound, C_16_H_14_Cl_2_O_3_, the dihedral angle between the mean planes of the two benzene rings is 55.30 (5)°. The methyl ester group lies within the ring plane due to an intra­molecular O—H⋯O hydrogen bond [maximum deviation from the C_8_O_2_ mean plane is 0.0383 (13) Å]. In the crystal, mol­ecules are held together by rather weak C—H⋯O hydrogen bonds.

## Related literature

For pharmacological relevance of salicylates and the synthesis of the title compound, see: Adeel, Rashid *et al.* (2009 ▶). For related structures, see: Adeel, Ali *et al.* (2009 ▶); Adeel, Langer *et al.* (2011 ▶).
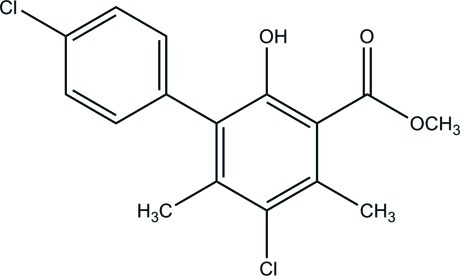


## Experimental

### 

#### Crystal data

C_16_H_14_Cl_2_O_3_
*M* *_r_* = 325.19
Monoclinic, 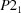

*a* = 4.0956 (5) Å
*b* = 13.3066 (17) Å
*c* = 13.3656 (16) Å
β = 92.711 (7)°
*V* = 727.59 (16) Å^3^
*Z* = 2
Mo *K*α radiation
μ = 0.45 mm^−1^
*T* = 173 K
0.65 × 0.50 × 0.06 mm

#### Data collection

Bruker APEXII CCD area-detector diffractometer
Absorption correction: multi-scan (*SADABS*; Bruker, 2005 ▶) *T*_min_ = 0.758, *T*_max_ = 0.973
9311 measured reflections
3754 independent reflections
3331 reflections with *I* > 2σ(*I*)
*R*_int_ = 0.022

#### Refinement

*R*[*F* ^2^ > 2σ(*F* ^2^)] = 0.030
*wR*(*F* ^2^) = 0.074
*S* = 1.04
3754 reflections
197 parameters
1 restraint
H atoms treated by a mixture of independent and constrained refinement
Δρ_max_ = 0.32 e Å^−3^
Δρ_min_ = −0.18 e Å^−3^
Absolute structure: Flack (1983 ▶), 1569 Friedel pairs
Flack parameter: 0.05 (5)

### 

Data collection: *APEX2* (Bruker, 2003 ▶); cell refinement: *SAINT* (Bruker, 2003 ▶); data reduction: *SAINT*; program(s) used to solve structure: *SHELXS97* (Sheldrick, 2008 ▶); program(s) used to refine structure: *SHELXL97* (Sheldrick, 2008 ▶); molecular graphics: *SHELXTL* (Sheldrick, 2008 ▶); software used to prepare material for publication: *SHELXTL*.

## Supplementary Material

Crystal structure: contains datablock(s) I, global. DOI: 10.1107/S1600536812012676/pv2517sup1.cif

Structure factors: contains datablock(s) I. DOI: 10.1107/S1600536812012676/pv2517Isup2.hkl

Supplementary material file. DOI: 10.1107/S1600536812012676/pv2517Isup3.cml

Additional supplementary materials:  crystallographic information; 3D view; checkCIF report

## Figures and Tables

**Table 1:** Hydrogen-bond geometry (Å, °)

*D*—H⋯*A*	*D*—H	H⋯*A*	*D*⋯*A*	*D*—H⋯*A*
O3—H3*O*⋯O1	0.77 (3)	1.80 (3)	2.523 (2)	156 (3)
C12—H12⋯O3^i^	0.95	2.53	3.306 (2)	139

Symmetry code: (i) 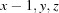.

**Fig. 1.**
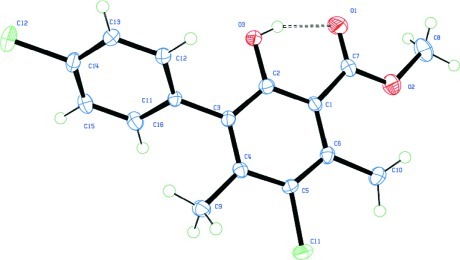
The molecular structure of the title compound with the atom numbering scheme. Displacement ellipsoids are drawn at the 50% probability level. H atoms are presented as small spheres of arbitrary radius.

**Fig. 2.**
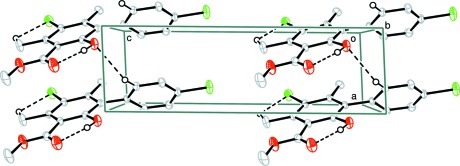
A view of the intermolecular (C—-H···O) and intramolecular (O—H···O and C—H···Cl) hydrogen bonds (dotted lines) in the crystal structure of the title compound. H atoms non-participating in hydrogen- bonding were omitted for clarity.

## supplementary crystallographic information

### Comment

Functionalized biaryls containing a 3-arylsalicylate substructure occur in a variety of pharmacologically relevant natural products (Adeel, Rashid & *et al.*, 2009). A sterically encumbered and functionalized biaryl, the title compound, was synthesized from 4-(4-methoxyphenyl)-1,3-bis(trimethylsilyloxy)-1,3-butadiene. In this paper, the crystal structure of the title compound has been presented.

In the title compound (Fig. 1), the dihedral angle between the mean planes of the two benzene rings is 55.30 (5)°. The methyl ester group lies within the ring plane due to an intramolecular O—H···O hydrogen bond; the maximum deviation of any atom from the mean-plane of atoms C1–C8/O1/O2 is 0.0383 (13) Å for C2. In the crystal, molecules are held together by rather weak intermolecular C—H···O hydrogen bonds along the *a*-axis (Fig. 2 & Table 1).

### Experimental

The title compound was prepared according to a previously published procedure (Adeel, Rashid *et al.*, 2009) using 3-(Silyloxy)-2-en-1-ones (332 mg, 1.65 mmol), 1,3-bis(silyl enol ethers) (612 mg, 1.65 mmol) and TiCl_4_ (0.18 ml, 1.65 mmol) in CH_2_Cl_2_ (4 mL). The title compound was isolated as a colorless prisms; (190 mg, 40%, m.p. = 367–369 K). Crystallization from a saturated dichloromethane/methanol (9:1) solution at ambient temperature gave colourless crystals suitable for X-ray crystallographic studies.

### Refinement

An absolute structure was determined by using 1569 Friedel pairs. The H atom bonded to O1 was located from a difference Fourier map and refined freely. Other H atoms were positioned geometrically and refined using a riding model, with C—H = 0.98 (methyl) or 0.95 Å (aryl) with *U*_iso_(H) = 1.5 times *U*_eq_(C) (methyl H) or 1.2 times *U*_eq_(C) (aryl H); torsion angles of all methyl groups were allowed to refine.

### Crystal data

**Table:**

C_16_H_14_Cl_2_O_3_	*F*(000) = 336
*M**_r_* = 325.19	*D*_x_ = 1.484 Mg m^−^^3^
Monoclinic, *P*2_1_	Mo *K*α radiation, λ = 0.71073 Å
Hall symbol: P 2yb	Cell parameters from 4567 reflections
*a* = 4.0956 (5) Å	θ = 2.2–29.8°
*b* = 13.3066 (17) Å	µ = 0.45 mm^−^^1^
*c* = 13.3656 (16) Å	*T* = 173 K
β = 92.711 (7)°	Plate, colourless
*V* = 727.59 (16) Å^3^	0.65 × 0.50 × 0.06 mm
*Z* = 2	

### Data collection

**Table:**

Bruker APEXII CCD area-detector diffractometer	3754 independent reflections
Radiation source: fine-focus sealed tube	3331 reflections with *I* > 2σ(*I*)
Graphite monochromator	*R*_int_ = 0.022
φ and ω scans	θ_max_ = 29.9°, θ_min_ = 3.1°
Absorption correction: multi-scan (*SADABS*; Bruker, 2005)	*h* = −5→5
*T*_min_ = 0.758, *T*_max_ = 0.973	*k* = −18→15
9311 measured reflections	*l* = −17→18

### Refinement

**Table:**

Refinement on *F*^2^	Secondary atom site location: difference Fourier map
Least-squares matrix: full	Hydrogen site location: inferred from neighbouring sites
*R*[*F*^2^ > 2σ(*F*^2^)] = 0.030	H atoms treated by a mixture of independent and constrained refinement
*wR*(*F*^2^) = 0.074	*w* = 1/[σ^2^(*F*_o_^2^) + (0.0402*P*)^2^ + 0.0613*P*] where *P* = (*F*_o_^2^ + 2*F*_c_^2^)/3
*S* = 1.04	(Δ/σ)_max_ < 0.001
3754 reflections	Δρ_max_ = 0.32 e Å^−^^3^
197 parameters	Δρ_min_ = −0.18 e Å^−^^3^
1 restraint	Absolute structure: Flack (1983), 1569 Friedel pairs
Primary atom site location: structure-invariant direct methods	Flack parameter: 0.05 (5)

### Special details

**Table:**

Geometry. All e.s.d.'s (except the e.s.d. in the dihedral angle between two l.s. planes) are estimated using the full covariance matrix. The cell e.s.d.'s are taken into account individually in the estimation of e.s.d.'s in distances, angles and torsion angles; correlations between e.s.d.'s in cell parameters are only used when they are defined by crystal symmetry. An approximate (isotropic) treatment of cell e.s.d.'s is used for estimating e.s.d.'s involving l.s. planes.
Refinement. Refinement of *F*^2^ against ALL reflections. The weighted *R*-factor *wR* and goodness of fit *S* are based on *F*^2^, conventional *R*-factors *R* are based on *F*, with *F* set to zero for negative *F*^2^. The threshold expression of *F*^2^ > σ(*F*^2^) is used only for calculating *R*-factors(gt) *etc*. and is not relevant to the choice of reflections for refinement. *R*-factors based on *F*^2^ are statistically about twice as large as those based on *F*, and *R*- factors based on ALL data will be even larger.

### Fractional atomic coordinates and isotropic or equivalent isotropic displacement parameters (Å^2^)

**Table:**

	*x*	*y*	*z*	*U*_iso_*/*U*_eq_	
Cl1	−0.12949 (11)	0.03726 (3)	0.31348 (3)	0.02951 (11)	
Cl2	−0.24707 (13)	0.25736 (5)	−0.35858 (3)	0.04068 (13)	
O1	0.4723 (4)	0.47523 (11)	0.24494 (11)	0.0385 (3)	
O2	0.4797 (4)	0.39445 (11)	0.39014 (10)	0.0397 (4)	
O3	0.1939 (3)	0.40131 (10)	0.08937 (10)	0.0257 (3)	
H3O	0.278 (6)	0.437 (2)	0.1272 (19)	0.046 (8)*	
C1	0.2187 (4)	0.31271 (13)	0.24869 (12)	0.0179 (3)	
C2	0.1324 (4)	0.31873 (13)	0.14433 (12)	0.0187 (3)	
C3	−0.0198 (3)	0.23718 (12)	0.09202 (11)	0.0167 (3)	
C4	−0.1037 (4)	0.15145 (12)	0.14643 (12)	0.0168 (3)	
C5	−0.0227 (4)	0.14812 (13)	0.25056 (12)	0.0182 (3)	
C6	0.1376 (4)	0.22493 (13)	0.30390 (12)	0.0190 (3)	
C7	0.3994 (4)	0.40092 (14)	0.29314 (13)	0.0219 (3)	
C8	0.6450 (6)	0.48150 (18)	0.43503 (17)	0.0449 (5)	
H8A	0.8541	0.4918	0.4037	0.067*	
H8B	0.6846	0.4701	0.5070	0.067*	
H8C	0.5076	0.5412	0.4245	0.067*	
C9	−0.2826 (4)	0.06380 (13)	0.09765 (13)	0.0218 (3)	
H9A	−0.3475	0.0810	0.0282	0.033*	
H9B	−0.4779	0.0486	0.1345	0.033*	
H9C	−0.1388	0.0049	0.0985	0.033*	
C10	0.2165 (5)	0.21357 (15)	0.41504 (13)	0.0309 (4)	
H10A	0.1166	0.1517	0.4391	0.046*	
H10B	0.1299	0.2714	0.4507	0.046*	
H10C	0.4540	0.2102	0.4273	0.046*	
C11	−0.0770 (4)	0.24329 (12)	−0.01993 (11)	0.0173 (3)	
C12	−0.2543 (4)	0.32261 (13)	−0.06551 (12)	0.0192 (3)	
H12	−0.3402	0.3741	−0.0251	0.023*	
C13	−0.3060 (4)	0.32687 (14)	−0.16939 (12)	0.0221 (3)	
H13	−0.4280	0.3805	−0.1995	0.026*	
C14	−0.1769 (4)	0.25169 (15)	−0.22847 (12)	0.0225 (3)	
C15	0.0039 (4)	0.17324 (14)	−0.18631 (13)	0.0235 (3)	
H15	0.0942	0.1231	−0.2274	0.028*	
C16	0.0510 (4)	0.16929 (13)	−0.08213 (13)	0.0221 (3)	
H16	0.1725	0.1152	−0.0526	0.026*	

### Atomic displacement parameters (Å^2^)

**Table:**

	*U*^11^	*U*^22^	*U*^33^	*U*^12^	*U*^13^	*U*^23^
Cl1	0.0416 (2)	0.02195 (19)	0.0246 (2)	−0.00629 (19)	−0.00240 (16)	0.00732 (18)
Cl2	0.0571 (3)	0.0497 (3)	0.01488 (19)	−0.0008 (3)	−0.00219 (17)	−0.0013 (2)
O1	0.0570 (9)	0.0258 (7)	0.0317 (7)	−0.0156 (6)	−0.0074 (6)	−0.0003 (6)
O2	0.0599 (9)	0.0338 (8)	0.0244 (7)	−0.0174 (7)	−0.0105 (6)	−0.0054 (6)
O3	0.0398 (7)	0.0188 (6)	0.0184 (6)	−0.0080 (5)	−0.0001 (5)	0.0008 (5)
C1	0.0206 (7)	0.0157 (8)	0.0173 (7)	0.0005 (6)	−0.0005 (6)	−0.0032 (6)
C2	0.0214 (7)	0.0175 (8)	0.0174 (7)	0.0018 (6)	0.0028 (6)	0.0005 (6)
C3	0.0175 (7)	0.0176 (8)	0.0148 (7)	0.0043 (6)	−0.0007 (5)	−0.0005 (6)
C4	0.0163 (6)	0.0159 (7)	0.0180 (7)	0.0027 (6)	−0.0002 (5)	−0.0014 (6)
C5	0.0217 (7)	0.0160 (7)	0.0168 (7)	0.0020 (6)	−0.0007 (6)	0.0038 (6)
C6	0.0192 (6)	0.0222 (8)	0.0152 (7)	0.0035 (6)	−0.0017 (5)	−0.0009 (6)
C7	0.0231 (8)	0.0214 (8)	0.0212 (8)	0.0000 (6)	−0.0005 (6)	−0.0049 (6)
C8	0.0619 (14)	0.0381 (13)	0.0331 (11)	−0.0169 (10)	−0.0135 (10)	−0.0141 (10)
C9	0.0226 (7)	0.0184 (8)	0.0239 (8)	−0.0016 (6)	−0.0039 (6)	0.0010 (6)
C10	0.0473 (10)	0.0283 (10)	0.0163 (8)	−0.0045 (8)	−0.0079 (7)	0.0034 (7)
C11	0.0189 (7)	0.0181 (8)	0.0148 (7)	−0.0018 (6)	−0.0013 (5)	−0.0002 (6)
C12	0.0221 (7)	0.0186 (8)	0.0167 (7)	0.0010 (6)	0.0012 (6)	−0.0007 (6)
C13	0.0274 (8)	0.0201 (8)	0.0184 (8)	0.0007 (6)	−0.0023 (6)	0.0025 (6)
C14	0.0237 (7)	0.0297 (9)	0.0139 (7)	−0.0046 (7)	−0.0002 (5)	−0.0003 (7)
C15	0.0238 (7)	0.0256 (9)	0.0211 (8)	0.0011 (7)	0.0010 (6)	−0.0083 (7)
C16	0.0205 (7)	0.0222 (9)	0.0231 (8)	0.0030 (6)	−0.0028 (6)	−0.0036 (7)

### Geometric parameters (Å, º)

**Table:**

Cl1—C5	1.7633 (17)	C8—H8B	0.9800
Cl2—C14	1.7507 (16)	C8—H8C	0.9800
O1—C7	1.225 (2)	C9—H9A	0.9800
O2—C7	1.325 (2)	C9—H9B	0.9800
O2—C8	1.457 (2)	C9—H9C	0.9800
O3—C2	1.352 (2)	C10—H10A	0.9800
O3—H3O	0.77 (3)	C10—H10B	0.9800
C1—C2	1.425 (2)	C10—H10C	0.9800
C1—C6	1.429 (2)	C11—C12	1.404 (2)
C1—C7	1.496 (2)	C11—C16	1.406 (2)
C2—C3	1.419 (2)	C12—C13	1.396 (2)
C3—C4	1.404 (2)	C12—H12	0.9500
C3—C11	1.506 (2)	C13—C14	1.394 (2)
C4—C5	1.416 (2)	C13—H13	0.9500
C4—C9	1.509 (2)	C14—C15	1.384 (3)
C5—C6	1.393 (2)	C15—C16	1.398 (2)
C6—C10	1.513 (2)	C15—H15	0.9500
C8—H8A	0.9800	C16—H16	0.9500
			
C7—O2—C8	116.13 (16)	C4—C9—H9B	109.5
C2—O3—H3O	104 (2)	H9A—C9—H9B	109.5
C2—C1—C6	119.90 (14)	C4—C9—H9C	109.5
C2—C1—C7	116.10 (15)	H9A—C9—H9C	109.5
C6—C1—C7	123.99 (14)	H9B—C9—H9C	109.5
O3—C2—C3	116.27 (14)	C6—C10—H10A	109.5
O3—C2—C1	122.25 (15)	C6—C10—H10B	109.5
C3—C2—C1	121.47 (15)	H10A—C10—H10B	109.5
C4—C3—C2	118.60 (14)	C6—C10—H10C	109.5
C4—C3—C11	121.88 (14)	H10A—C10—H10C	109.5
C2—C3—C11	119.49 (14)	H10B—C10—H10C	109.5
C3—C4—C5	118.87 (14)	C12—C11—C16	118.01 (14)
C3—C4—C9	121.99 (14)	C12—C11—C3	121.66 (14)
C5—C4—C9	119.13 (15)	C16—C11—C3	120.33 (14)
C6—C5—C4	124.26 (15)	C13—C12—C11	120.89 (15)
C6—C5—Cl1	119.44 (12)	C13—C12—H12	119.6
C4—C5—Cl1	116.29 (13)	C11—C12—H12	119.6
C5—C6—C1	116.81 (14)	C14—C13—C12	119.38 (15)
C5—C6—C10	120.19 (15)	C14—C13—H13	120.3
C1—C6—C10	123.00 (14)	C12—C13—H13	120.3
O1—C7—O2	120.77 (16)	C15—C14—C13	121.36 (15)
O1—C7—C1	123.58 (16)	C15—C14—Cl2	119.75 (13)
O2—C7—C1	115.66 (15)	C13—C14—Cl2	118.88 (14)
O2—C8—H8A	109.5	C14—C15—C16	118.68 (15)
O2—C8—H8B	109.5	C14—C15—H15	120.7
H8A—C8—H8B	109.5	C16—C15—H15	120.7
O2—C8—H8C	109.5	C15—C16—C11	121.67 (15)
H8A—C8—H8C	109.5	C15—C16—H16	119.2
H8B—C8—H8C	109.5	C11—C16—H16	119.2
C4—C9—H9A	109.5		
			
C6—C1—C2—O3	177.81 (15)	C2—C1—C6—C10	−179.09 (15)
C7—C1—C2—O3	−3.4 (2)	C7—C1—C6—C10	2.3 (2)
C6—C1—C2—C3	−3.0 (2)	C8—O2—C7—O1	2.3 (3)
C7—C1—C2—C3	175.71 (14)	C8—O2—C7—C1	−177.79 (17)
O3—C2—C3—C4	−176.93 (14)	C2—C1—C7—O1	0.1 (2)
C1—C2—C3—C4	3.9 (2)	C6—C1—C7—O1	178.84 (16)
O3—C2—C3—C11	5.0 (2)	C2—C1—C7—O2	−179.73 (14)
C1—C2—C3—C11	−174.17 (14)	C6—C1—C7—O2	−1.0 (2)
C2—C3—C4—C5	−2.3 (2)	C4—C3—C11—C12	125.94 (16)
C11—C3—C4—C5	175.72 (14)	C2—C3—C11—C12	−56.1 (2)
C2—C3—C4—C9	176.59 (14)	C4—C3—C11—C16	−54.8 (2)
C11—C3—C4—C9	−5.4 (2)	C2—C3—C11—C16	123.15 (16)
C3—C4—C5—C6	−0.2 (2)	C16—C11—C12—C13	1.0 (2)
C9—C4—C5—C6	−179.04 (14)	C3—C11—C12—C13	−179.75 (14)
C3—C4—C5—Cl1	−178.97 (11)	C11—C12—C13—C14	−0.6 (2)
C9—C4—C5—Cl1	2.1 (2)	C12—C13—C14—C15	−0.5 (2)
C4—C5—C6—C1	1.0 (2)	C12—C13—C14—Cl2	179.39 (13)
Cl1—C5—C6—C1	179.78 (12)	C13—C14—C15—C16	1.2 (2)
C4—C5—C6—C10	−179.32 (16)	Cl2—C14—C15—C16	−178.73 (13)
Cl1—C5—C6—C10	−0.5 (2)	C14—C15—C16—C11	−0.8 (2)
C2—C1—C6—C5	0.6 (2)	C12—C11—C16—C15	−0.3 (2)
C7—C1—C6—C5	−178.07 (15)	C3—C11—C16—C15	−179.57 (14)

### Hydrogen-bond geometry (Å, º)

**Table:**

*D*—H···*A*	*D*—H	H···*A*	*D*···*A*	*D*—H···*A*
O3—H3*O*···O1	0.77 (3)	1.80 (3)	2.523 (2)	156 (3)
C12—H12···O3^i^	0.95	2.53	3.306 (2)	139
C10—H10*A*···Cl1	0.98	2.45	3.029 (2)	118

Symmetry code: (i) *x*−1, *y*, *z*.
